# The Prolific Ternary System Pt/Sn/Nd: Insertion of Pt into the Structures of Sn/Nd Intermetallics Yields Structural Complexity and Wealth

**DOI:** 10.1021/acs.inorgchem.3c00318

**Published:** 2023-06-02

**Authors:** Chris Celania, Volodymyr Smetana, Gerd H. Meyer, Anja-Verena Mudring

**Affiliations:** † Department of Materials and Environmental Chemistry, Stockholm University, Svante Arrhenius väg 16 C, 10691 Stockholm, Sweden; ‡ intelligent Advanced Materials, Department of Biological and Chemical Engineering and iNANO, Aarhus University, 8000 Aarhus C, Denmark; § Department of Chemistry, Universität zu Köln, Greinstraße 6, 50939 Köln, Germany; ∥ Department of Chemistry, Royal Institute of Technology (KTH), Teknikringen 26, 10042 Stockholm, Sweden

## Abstract

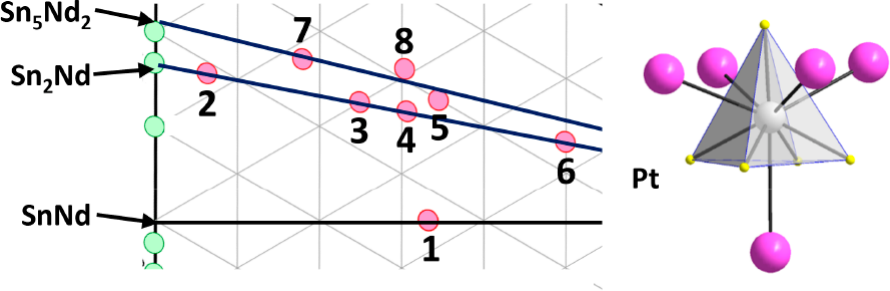

The understanding of structure and bonding in intermetallic phases still lags behind that of molecular compounds. For that reason, exploring intermetallic phases and identifying structural patterns and relationships are particularly important for closing this knowledge gap. In particular, here we report on the addition of increasing amounts of platinum to ∼2:1 mixtures of tin and neodymium, which yields eight ternary Pt/Sn/Nd compounds, four of which have not been reported before. Interestingly, except for PtSnNd (**1**), all observed ternary phases of the system can be derived from the binary compounds Sn_2_Nd and Sn_5_Nd_2_ by adding Pt to the composition(s), as they lie on or close to two lines: Sn_2_Nd–Pt (Pt_0.21(1)_Sn_2_Nd (**2**), PtSn_2_Nd (**3**), Pt_1.33_Sn_2_Nd (**4**), Pt_2–*x*_Sn_2+*x*_Nd (*x* = 0.27(3), **5**), and Pt_3_Sn_2_Nd (**6**)) or Sn_5_Nd_2_–Pt (Pt_1.5_Sn_5–*x*_Nd_2_ (*x* = 0.16(2), **7**) and Pt_3_Sn_5_Nd_2–*x*_ (*x* = 0.161(8), **8**)). While the introduction of increasing amounts of Pt to the binaries Sn_2_Nd and Sn_5_Nd_2_ leads to stepwise changes in the coordination environment of Nd, Pt preserves its coordination over the entire system in the form of interpenetrating bipyramidal {PtSn_5_Nd_5_} clusters.

## Abstract 2

A systematic exploration of the Nd−Pt−Sn ternary system revealed a stunning wealth of compounds. Seven of the eight compounds observed in the ternary system Pt/Sn/Nd can even be structurally derived from the binary compounds Sn_2_Nd and Sn_5_Nd_2_ by adding Pt.

## Introduction

In contrast to molecular and coordination compounds, the chemical bonding and structure in extended solids is less intuitive to understand and, unlike for the former, not easy to predict. While for simple salts predictive rules based on ionic radii exist, these start to fail once the differences in electronegativity start to vanish and simple assignment of charges and oxidation states are no longer possible. Particularly interesting is to see the changes that occur when moving from salt-like rare-earth metal halides, to rare-earth metal cluster complex halides, to intermetallics.

While rare-earth metal compounds frequently feature the rare earth metal R in the oxidation state +III, it is possible to realize oxidation states below that for R in rare-earth metal (R = Sc, Y, La, Ce–Lu) cluster complex halides (X = Cl, Br, I). In almost all cases they require an endohedral (interstitial) atom Z, either from a transition metal T or a post-transition p-element E including hydrogen.^1^ Small groups like C_2_, BC, or CBC may also be encapsulated.^2^ Interestingly, the structure and bonding in these compounds bears strong analogies to Werner-type coordination complexes. As the endohedral atom, albeit being negatively charged, has the same function as the commonly positively charged central atom in a Werner-type coordination complex, they have therefore been coined as *anti*-Werner complexes,^3^ and it is reasonable to write cluster complex halide formulas as {Z_*z*_R_*r*_}X_*x*_, for example {PtY_6_}I_10_.^4,5^

Typically, such cluster complex halides are prepared by reacting the respective transition metal T such as platinum together with the respective rare-earth metal R and one of its halides, RX_3_, which serves as a reactive self-flux. As the halide’s atomic number decreases, competition increases between forming the cluster complex halide {T_*t*_R_*r*_}X_*x*_ and the binary intermetallic, {T_*t*_R_*r*_}, where the rare-earth metal halide acts as a nonreacting, innocent flux. For example, Pt_3_Pr_4_ was obtained during an attempt to synthesize {PtPr_3_}Cl_3_.^6^ Within this same study, an even more innocent flux, molten NaCl, produced the new Pt_1.97_Pr_3_ with a defect Ga_2_Gd_3_ type of structure.

The reactive metal flux tin, however, led to three new ternary intermetallics with tin, Pt_4_Sn_6_Pr_2.91_, Pt_4_Sn_6_Pr_3_, and Pt_12_Sn_24_Pr_4.84_.^6^ The only ternary intermetallic that had previously been observed in the Pt/Sn/Pr system is PtSnPr, which adopts the TiNiSi type of structure^7^ at ambient pressure (NP-PtSnPr) and the ZrNiAl type at 10.5 GPa (HP-PtSnPr).^8^ In a subsequent study, analogous to Pt_4_Sn_6_Pr_3_, similar intermetallics crystallizing with the Pt_4_Ge_6_Pr_3_-type of structure^9^ doubtlessly were synthesized with R = La and Ce, but there were doubts about Pt_4_Sn_6_Nd_3_. However, for the Pt/Sn/Nd system, Pt_3_Sn_5_Nd_1.84_ was observed, crystallizing with a defect Rh_3_Sn_5_Y_2_ type arrangement^10^ together with PtSnNd^11^ and Pt_3_Sn_2_Nd.^12^ It is also worth noting that despite frequently this and similar systems get addressed as stannides, all compounds in this system are platinides, as Pt is the element with the highest electronegativity, which is supported by theoretical calculations.^10^

Continuing our previous investigations of the related systems with other light lanthanides, particularly isostructural rows in those systems,^6,10^ we focused here on the system with Nd and explored the phase space along specific directions. The lower melting tin-rich part of the binary system Sn/Nd contains two congruently melting intermetallic compounds, Sn_2_Nd (1180 °C, 66.7 mol % Sn) and Sn_3_Nd (1150 °C, 75 mol % Sn), encapsulating not only a eutectic but also two incongruently melting compounds, Sn_7_Nd_3_ and Sn_5_Nd_2_.^13^ While Sn_3_Nd crystallizes with the Cu_3_Au type of structure, the other three crystallize with ordered three- (Sn_2_Nd), five- (Sn_7_Nd_3_), and sevenfold (Sn_5_Nd_2_) superstructures of that type. The addition of platinum to ∼2:1 mixtures of tin and neodymium at reaction temperatures at and below 1000 °C, with or without the use of NaCl as a nonreactive, innocent flux or tin acting as a reactive self-flux, produced eight ternary Pt/Sn/Nd compounds, revealing intriguing insights into compositional and structural relationships with the binary Sn/Nd system.

## Experimental Section

High-purity starting materials were obtained from the following locations: neodymium (Hefa Rare Earth Canada, 99.8%), platinum (Alfa Aesar 99.95%), and tin (Alfa Aesar, 99.999%). To reduce possible undesired impurities originating from surface contaminations, the top layer of neodymium was filed away to remove the surface oxide, and all handling and loading of starting materials took place in an argon-filled glovebox.

As this work originally began in an attempt to corroborate results of a potential Pt_4_Sn_6_Nd_3_ phase, the first sample was started from a Pt/Sn/Nd = 4:6:3 molar mixture loaded to a total mass of 0.5 g into a tantalum ampule under argon and arc welded before being flame-sealed within a fused silica tube under vacuum. The sample was placed in a box furnace and heated at 1000 °C for 24 h before being cooled to 900 °C and held for four days. The sample was then cooled to room temperature by switching off the power to the furnace. This sample contained single crystals of three ternary intermetallics according to
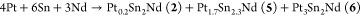
1

**Figure 1:**
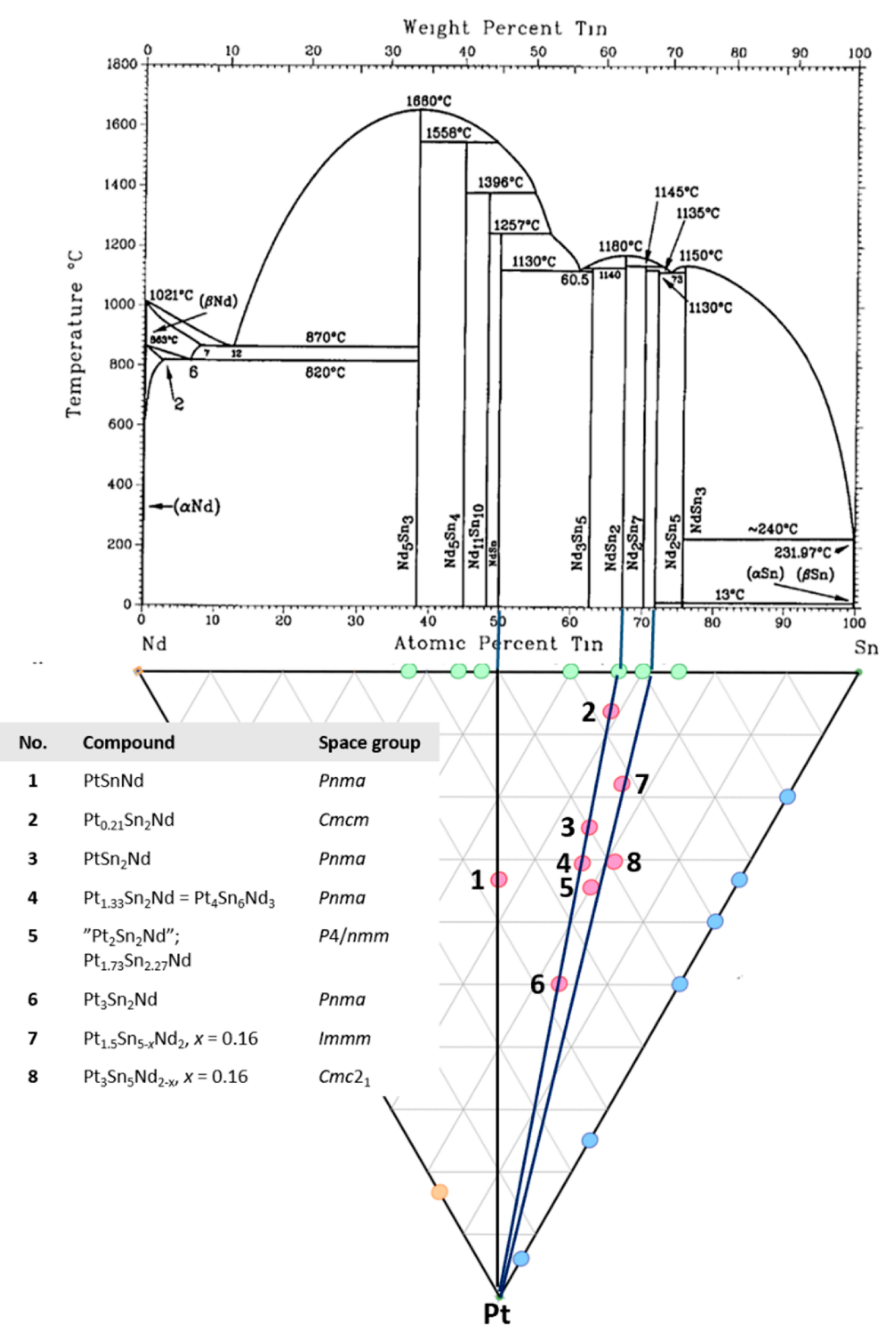
(Top) Phase diagram of the binary system Nd/Sn. Reproduced with permission from ref (13). Copyright 1994 Springer Nature. (Bottom) The Pt/Sn/Nd compound triangle with, at present, eight ternary intermetallics, five of which lie on or close to the Sn_2_Nd–Pt section, **2**–**6**, and two close to the Sn_5_Nd_2_–Pt section (**7** and **8**).

For the numbering scheme of all compounds, please consult Figure 1. Please note, this and further reaction schemes are just to show where single crystals of the new compounds were observed and do not play the part of the reaction equations. The products could also contain an excess of Sn and possibly minor amounts of additional unidentified phases.

The next sample was loaded at the same starting composition (4:6:3), but with an additional 0.25 g of dried NaCl to be used as a fluxing agent. The sample was held at 1000 °C for 72 h and then furnace cooled to 900 °C for a shorter anneal of only 24 h before being furnace cooled back to room temperature. Single crystals of two ternaries were detected during exploration for single crystal X-ray diffraction (SCXRD) of this sample.
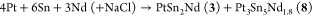
2

Additionally, a sample loaded at a 1:2:1 starting composition with NaCl flux was also created using lower temperatures. The 1:2:1 + NaCl sample was heated to 900 °C for only 5 h, then slowly cooled at 10 °C/h to 500 °C, where it was annealed for 72 h before cooling in the furnace to room temperature, leading to the following result:
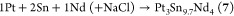
3

Finally, a 0.5 g sample with a starting composition of Pt/Sn/Nd = 0.21:2:1 (targeting for **2**) was loaded, pelletized in a die press, prereacted up to 600 °C for 1 h before being removed and arc melted. The sample was subsequently flipped and remelted four times to encourage full homogenization. PXRD confirmed the sample to be phase-pure directly after arc melting. Thus, an additional anneal was not performed.

X-ray powder diffraction for phase analysis was performed on samples using a PANalytical X’Pert Pro diffractometer with Cu Kα_1_ radiation (λ = 1.54059 Å). For powder preparation, the brittle samples were ground manually with an agate mortar and pestle and dispersed onto a thin layer of vacuum grease on top of a single crystal silicon plate. Data analysis for phase assignment was performed with the WinXPow 3.06 software package.^14^

Single crystals were selected under an optical microscope from manually crushed samples and then affixed to a glass fiber with vacuum grease. Single-crystal X-ray diffraction was performed at room temperature on a Bruker D8 VENTURE diffractometer (Mo Kα radiation; λ = 0.71073 Å), and the APEX3 software suite was utilized for data collection, integration, polarization, and empirical absorption correction.^15,16^ Scans ranged 2θ values of ∼4–66°. APEX3 was also utilized to check for extinction conditions and *E* statistics in the intensity data sets required for proper space group assignment, as well as to carry out full structure refinement, (i.e., refining atomic positions, determining mixed site occupancies, identifying disordered positions, and anisotropic displacement parameters).^17,18^

Single-crystal refinement data for the new compounds are summarized in Table 1, which displays the relevant refinement information and parameters. Site-specific positional information for the compounds may be deduced from data deposited with CCDC as .cif files.

**Table 1:** Crystallographic Details and Refinement Parameters for Pt_0.21(1)_Sn_2_Nd (**2**), PtSn_2_Nd (**3**), Pt_1.73(3)_Sn_2.27(3)_Nd (**5**), and Pt_3_Sn_9.67(3)_Nd_4_ (**5**)

parameter	Pt_0.21(1)_Sn_2_Nd (**2**)	PtSn_2_Nd (**3**)	Pt_1.73(3)_Sn_2.27(3)_Nd (**5**)	Pt_3_Sn_9.67(3)_Nd_4_ (**7**)
CCDC no.	1962954	1962957	1962955	1962958
formula weight, fw [g/mol]	421.78	576.71	750.87	2310.51
space group	*Cmcm*	*Pnma*	*P*4/*nmm*	*Immm*
*a* (Å)	4.5132(5)	16.392(2)	4.546(4)	4.5875(3)
*b* (Å)	16.853(2)	4.5937(6)	4.546(4)	9.2942 (6)
*c* (Å)	4.4246(5)	15.080(2)	10.409(11)	36.713(3)
*V* (Å^3^)	336.54(7)	1135.5(3)	215.1(4)	1565.34(18)
*Z*	4	12	2	4
radiation type	Mo Kα			
μ (mm^–1^)	38.13	63.11	80.58	54.78
data collected	1343	8658	2121	10042
intensity data/restraints/parameters	214/0/ 21	1265/0/74	154/0/ 18	1435/0/ 71
*R*_int_	0.021	0.084	0.055	0.037
index ranges	–5 < *h* < 5	–20 < *h* < 20	–5 < *h* < 5	–4 < *h* < 5
	–20 < *k* < 20	–5 < *k* < 5	–5 < *k* < 4	–11 < *k* < 10
	–5 < *l* < 5	–18 < *l* < 18	–12 < *l* < 12	–44 < *l* < 45
refinement method	Full-matrix least-squares on *F*^2^			
*R*_1_, ω*R*_2_ [*I*_0_ > 2σ(*I*)]	0.0165, 0.0330	0.0339, 0.0548	0.0383, 0.989	0.0301, 0.0796
*R*_1_, ω*R*_2_ (all data)	0.0187, 0.0336	0.0578, 0.0584	0.0386, 0.990	0.0317, 0.0804
Δρ_max_/Δρ_min_ (e/Å^3^)	1.39/ – 1.49	2.11/ – 2.66	4.23/ – 3.71	3.07/ – 2.81

### Microscopy Analysis

Compositional analyses for compounds **2** and **7** were performed with a JEOL 7000F scanning electron microscopy system equipped with Oxford Inca X-sight energy dispersive spectrometer (EDS) at an acceleration voltage of 20 kV. The samples in the form of single crystals or just larger fragments with relatively flat surfaces were mounted on a Bakelite. Microscopic examination were carried out in the back scattered electron mode. EDS optimization was done with a pure copper metal piece. Results of the EDS analysis are presented in Tables S1 and S2.

## Results and Discussion

The binary systems Nd/Sn, Pt/Sn, and Nd/Pt contain nine, six, and six binary compounds, respectively, totaling 21 altogether. Figure 1 shows the reported binary system Nd/Sn.^13^ Interestingly, except for PtSnNd, seven of the known eight compounds in the ternary system Pt/Sn/Nd may be derived from the binary compounds Sn_2_Nd and Sn_5_Nd_2_ by adding Pt to the composition(s), see Figure 1. This is particularly interesting as the melting point of Pt at 1772 °C significantly exceeds those of Nd (1021 °C) and Sn (232 °C),^19^ with the latter being the one first to melt. Although the Sn_2_Nd–Sn_5_Nd_2_–Pt triangle represents only a tiny fraction of the whole Pt/Sn/Nd phase space, it contains practically all so-far discovered compositions.

The three ternary intermetallics that we obtained via reaction (**1**), Pt_0.2_Sn_2_Nd (**2**), Pt_1.7_Sn_2.3_Nd (**5**), and Pt_3_Sn_2_Nd (**6**), have a molar Sn/Nd ratio of (or about) 2:1 with varied Pt content, the highest-melting metal by far, from 0.2 to 3. Additionally, in reactions (**2**) and (**3**), Sn/Nd starting ratios of 2:1 are at or not far from this ratio in PtSn_2_Nd (**3**), Pt_3_Sn_5_Nd_1.8_ (**8**), and Pt_3_Sn_9.7_Nd_4_ (**7**). Except for PtSnNd (**1**), the Sn/Nd ratio is between 2 and 2.78 for the other seven compounds. Thus, they are located either on or close to the Sn_2_Nd–Pt line (**2**, **3**, **4**, **5**, and **6**). Otherwise, they lie above in the tin-richer regime (**7** and **8**) on or about the Sn_5_Nd_2_–Pt section, see Figure 1. Table 2 gives an overview of the lattice constants and molar volumes of these compounds, including values for Sn_2_Nd and Sn_5_Nd_2_, data that are needed for the following discussion.

**Table 2:** Lattice Constants, Molar Volumes, and Average Distances for Intermetallics Related to Sn_2_Nd and Sn_5_Nd_2_

								*V*_m_/ [cm^3^/mol]					
	compound	ref.	space group	*a* (Å)	*b* (Å)	*c* (Å)	*Z*	obs.^a^	calc.^a^	Δ*V*_m_ (%)	⟨*d*⟩(Pt–Sn) [CN]^b^	⟨*d*⟩(Pt–Nd) [CN]	⟨*d*⟩(Nd–Sn) [CN]
**1**	PtSnNd	(11)	*Pnma*	7.372(1)	4.600(1)	7.996(2)	4	40.83	45.98	11.2	2.750(4)	3.278(6)	3.304(6)
	Sn_2_Nd	(20)	*Cmmm*	4.4404(8)	15.944(4)	4.5628(8)	4	48.64	53.16	8.5			3.453(10)
			*Cmcm*	4.342^c^	17.290	4.303	4						
**2**	Pt_0.2_Sn_2_Nd		*Cmcm*	4.5132(5)	16.853(2)	4.4246(5)	4	50.67	55.07	8.0	2.425(5)	3.516(5)	3.335(10)
**3**	PtSn_2_Nd		*Pnma*	16.392(2)	4.5937(6)	15.080(2)	12	56.99	62.16	8.3	1: 2.693(5)	1: 3.379(5)	1: 3.230(8)
											2: 2.672(5)	2: 3.415(5)	2: 3.380(9)
											3: 2.675(5)	3: 3.466(5)	3: 3.101(9)
											2.680^d^	3.420	3.237
**4**	Pt_4_Sn_6_Nd_3_ = Pt_1.33_Sn_2_Nd	(10)	*Pnma*	27.647(3)	4.5858(9)	9.326(1)	4	178.04			1: 2.632(5)	1: 3.547(5)	1: 3.490(9)
								59.35^e^	65.29	9.1	2: 2.669(5)	2: 3.427(5)	2: 3.507(9)
											3: 2.671(5)	3: 3.426(5)	3: 3.291(9)
											4: 2.647(5)	4: 3.632(5)	
											2.655	3.508	3.429
**5**	Pt_2-*x*_Sn_2+*x*_Nd *x* = 0.27		*P*4/*nmm*	4.546(4)	= *a*	10.409(11)	2	64.78	73.30	11.6	1: 2.648(5)	1: 3.464(4)	3.519(9)
									71.36^f^	9.2	2: 2.586(4)	2: 3.398(4)	
											2.617	3.431	
**6**	Pt_3_Sn_2_Nd	(12)	*Pnma*	9.6201(4)	4.7362(2)	10.3103(4)	4	70.73	80.46	12.1	1: 2.775(5)	1: 3.489(4)	3.398(7)
											2: 2.789(4)	2: 3.127(3)	
											3: 2.773(4)	3: 3.112(3)	
											2.779	3.243	
	Sn_5_Nd_2_	(20)	*Cmmm*	4.5688(6)	35.119(4)	4.6139(6)	4	111.47	122.60	9.1			1: 3.298(12)
													2: 3.333(10)
													3.316
**7**	Pt_1.5_Sn_4.84_Nd_2_		*Immm*	4.5875(3)	9.2941 (6)	36.713(3)	8	235.70	267.13	11.8	1: 2.683(5)	1: 3.369(5)	1: 3.209(8)^h^
								117.85^g^	133.57		2: 2.625(5)	2: 3.515(5)	4: 3.320(12)
											2.654	3.442	3.265
**8**	Pt_3_Sn_5_Nd_1.84_	(10)	*Cmc*2_1_	4.515(3)	26.14(2)	7.291(5)	4	129.57	145.78	11.1	1: 2.720(5)	1: 3.254(4)	1: 3.327(8)
											2: 2.799(6)	2: 3.172(3)	2: 3.331(10)
											3: 2.757(7)		
											2.759	3.213	3.329
overall averaged distances:											2.665	3.381	3.359
sum of the respective atomic radii:											2.981	3.209	3.416

a The observed molar volume is calculated via *V*_m_ = (*a·b·c*·*N*_A_)/*Z*; *V*_m_(obs.) is the sum of the respective atomic volumes, i.e., 9.10 (Pt), 16.28 (Sn), and 20.60 (Nd) cm^3^/mol.

b CN = coordination number, i.e., number of nearest neighbors.

c Values in this row are calculated for the same volume as observed for Sn_2_Nd but with the lattice constant ratios of nonexistent ZrSi_2_-type Sn_2_Nd.^26^

d Values in italics are the average over all positions.

e Volume divided by 3 to match the formula Pt_4/3_Sn_2_Nd.

f Calculated for Pt_2_Sn_2_Nd.

g Volume for *Z* = 2 for better comparison with Sn_5_Nd_2_ and **8**.

h Nd2–Sn and Nd3–Sn surroundings are affected by the under-occupation and disorder of Sn7–10.

### PtSnNd: The Only Ternary Intermetallic on the Pt–SnNd Line

Although the binary-phase SnNd is incorporated in the Sn/Nd phase diagram as a compound melting incongruently at 1257 °C,^13^ see Figure 1, it is also said to be unknown.^20^ Nevertheless, the intermetallic with a 1:1:1 composition, PtSnNd, lies on the SnNd–Pt line at 50 mol %. During our present study, we have only seen traces of PtSnNd in reaction (**1**). This is, of course, due to the fact that starting mixtures with a Sn/Nd ratio of 2:1 have been used, as we were originally targeting Pt_4_Sn_6_Nd_3_ = Pt_1.33_Sn_2_Nd. Furthermore, PtSnNd (**1**), usually addressed as a stannide, NdPtSn, has been well studied.^11^ It crystallizes with an *anti*-PbCl_2_ or TiNiSi type of structure,^7^ according to PbCl1Cl2 ≡ SiNiTi ≡ PtSnNd. Coordination numbers of all atoms are high, 10 for Pt (4 Sn + 6 Nd), 12 for Sn (4 Pt + 6 Nd + 2 Sn), and 16 for Nd (6 Pt, 6 Sn + 4 Nd), which suit the sizes of the atomic radii well: *r*(Pt) = 1.387 Å, *r*(Sn) = 1.594 Å, and *r*(Nd) = 1.822 Å.^21^ Average Pt–Sn and Nd–Sn distances are much shorter and somewhat shorter, respectively, than the respective sum of the atomic radii, while the Pt–Nd average distances are larger, see Table 2. This is a trend that we will see throughout the ternary compounds in the Pt/Sn/Nd system.

### Intermetallics on or Close to the Pt–Sn_2_Nd Line

**Figure 2:**
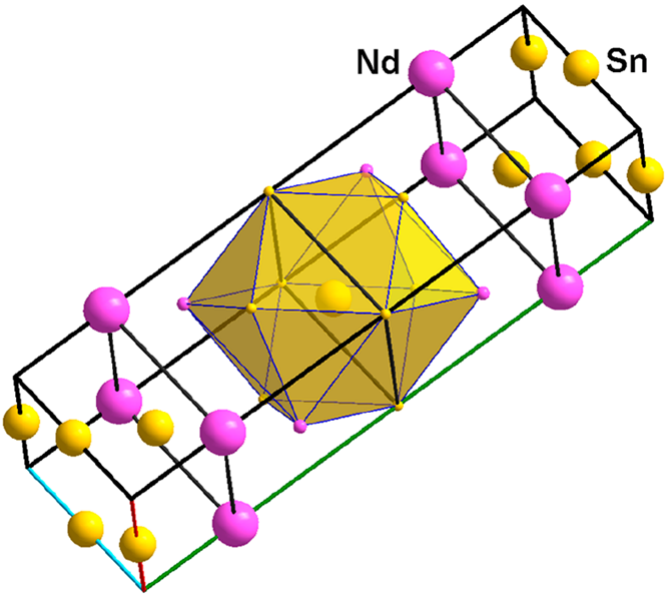
One unit cell of the crystal structure of Sn_2_Nd shows the threefold superstructure of a pseudo-face-centered unit cell and one dodecahedron, Sn3@Sn_8_Nd_4_. Crystallographic axes are color-coded as follows: *a*, red; *b*, green; and *c*, blue.

Platinum crystallizes with the cubic closest packed structure (ccp, fcc, A1) with *a* = 3.9237 (3) Å.^22^ Accordingly, it contains a shortest Pt–Pt distance of 2.774 (1) Å, which corresponds to an atomic radius *r*(Pt) of 1.387 Å for coordination number 12^21^ (a dodecahedron of Pt atoms surrounding each Pt atom). NdSn_2_ at the other end of the Pt–Sn_2_Nd line crystallizes with the ZrGa_2_/UGe_2_/NdSn_2_ type of structure.^20,23−25^ This is actually an elongated threefold superstructure of the cubic closest packed structure type with *a* = 4.4404 (8), *b* = 15.944 (4), *c* = 4.5628 (8) Å, and space group *Cmmm* (no. 65).^20^ All four crystallographically independent atom positions, Nd (4*i*), Sn1 (4*j*), Sn2 (2*b*), and Sn3 (2*d*), are surrounded by somewhat distorted dodecahedra of a mix of Sn and Nd atoms, Nd@Nd_2_Sn_10_ as well as Sn@Sn_8_Nd_4_, see Figure 2.

Between Pt and Sn_2_Nd we have found four compounds: Pt_0.2_Sn_2_Nd (**2**), PtSn_2_Nd (**3**), Pt_4/3_Sn_2_Nd = Pt_4_Sn_6_Nd_3_ (**4**), and Pt_3_Sn_2_Nd (**6**). Pt_1.7_Sn_2.3_Nd (**5**) would be on that line as Pt_2_Sn_2_Nd if there were no mixing of Pt and Sn.

#### Pt_0.21_Sn_2_Nd (**2**)

**Figure 3:**
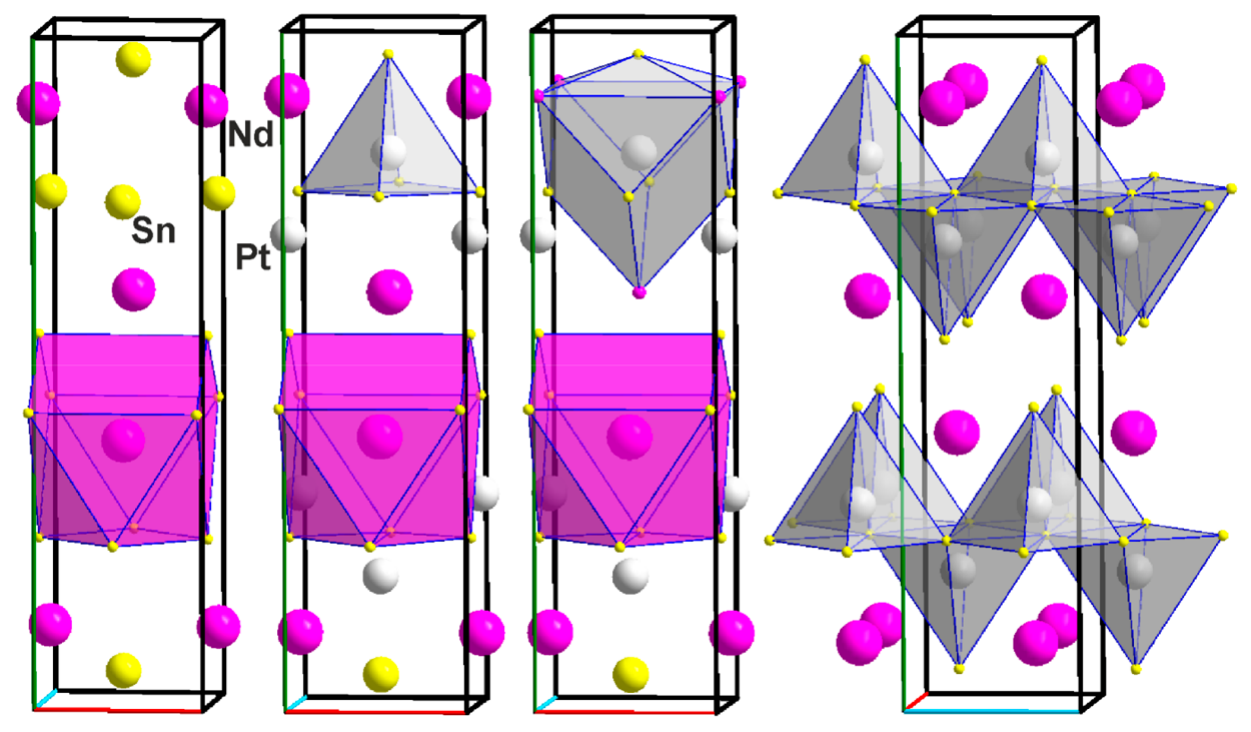
From left to right: crystal structure of the nonexistent ZrSi_2_-type form of Sn_2_Nd (= Si_2_Zr) in comparison with that of the stuffed derivative Pt_0.21_Sn_2_Nd (**2**) exhibiting the {PtSn_5_} square pyramids and {PtSn_5_Nd_5_} clusters, as well as the connection of the square pyramids {PtSn_5_}. Crystallographic axes are color-coded as follows: *a*, red; *b*, green; and *c*, blue.

Pt_0.21_Sn_2_Nd (**2**)crystallizes with a stuffed derivative of the nonexistent ZrSi_2_ type of structure of Sn_2_Nd, see Figure 3. The lattice constants of the orthorhombic unit cell of (**2**) compare well with the lattice constants of Sn_2_Nd (ZrGa_2_ type), with an elongated *b*-axis, as well as with those of ZrSi_2_^26^ with (**2**) having a considerably larger cell and, correspondingly, molar volume (Table 3).

**Table 3:** Comparison of the Unit Cells of **2**, **3**, and Binary Sn_2_Nd and ZrSi_2_

compound	*a* (Å)	*b* (Å)	*c* (Å)	*V*_*m*_ (cm^3^/mol)
Pt_0.21_Sn_2_Nd (**2**)	4.5132(5)	16.853(2)	4.4246(5)	50.67
PtSn_2_Nd (**3**)	4.5937(6)	16.392(2)	15.080(2)	56.99
Sn_2_Nd (ZrGa_2_ type)	4.4404(8)	15.944(4)	4.5628(8)	53.16
ZrSi_2_	3.706(1)	14.735(3)	3.672(1)	48.64

The volume contraction of (**2**) originates from bonding interactions in the intermetallic. The difference between the real volumes of Pt_0.2_Sn_2_Nd and Sn_2_Nd, 2.03 cm^3^/mol, compares surprisingly well with 21% of the atomic volume of Pt, 1.91 cm^3^/mol. The additional Pt atoms in the otherwise nonexistent ZrSi_2_ form of Sn_2_Nd occupy a position as depicted in Figure 3. They are surrounded by two interpenetrating square pyramids of Sn and Nd at distances of Pt–Sn between 2.3896 (8) and 2.4543 (9) Å with an average of 2.425 (1) Å, as well as Pt–Nd distances between 3.448 (2) and 3.5330 (9) Å with an average of 3.516 (1) Å. While the Pt–Nd distances are elongated with respect to the sum of the atomic radii, 3.209 Å, the Pt–Sn distances are considerably shorter than the sum of the atomic radii, 2.981 Å. This may be due to the under-occupation of the Pt site (only 21%), as we can see from a comparison with an overall averaged Pt–Sn distance of 2.665 (1) Å (see Table 2).

The reason for the under-occupation of the Pt site in Pt_0.21_Sn_2_Nd (**2**) is not clear, but it appears to be rather common for the BaCuSn_2_ (or CuSn_2_Ba) type of structure when rare-earth metals are involved, as in T_*x*_Sn_2_La with T = Fe (*x* = 0.34), T = Co (*x* = 0.52), T = Ni (*x* = 0.74), and T = Cu (*x* = 0.56).^27^ On the other hand, there is no hint in the literature about the (under-) occupancy of the Ni site in NiSi_2_Ce,^28^ which also exhibits the CuSn_2_Ba type of structure like Pt_0.21_Sn_2_Nd (**2**). This compound and a variety of transition-metal/group 14/rare-earth metal analogs have been investigated for their magnetic behavior.^29,30^ However, the occupancy of the *T* site is a feature that has not been fully investigated so far.

Except for the under-occupation of the Pt site, the crystal structure determination of Pt_0.21_Sn_2_Nd (**2**) shows 0.71:0.29 disorder on one of the Sn sites (Sn2a and Sn2b). For the drawing of the unit cell in Figure 3 the midpoint of the *x*/*a* parameters of 0.5651 and 0.5425, 0.554, was taken.

#### PtSn_2_Nd (**3**)

PtSn_2_Nd (**3**)crystallizes with the LuNiSi_2_ type of structure,^31^ PtSn_2_Nd ≡ NiSi_2_Lu. A comparison of the lattice constants and molar volumes as well as the number of formula units within the unit cells (Table 3) suggests a threefold “super-structure”. Indeed, the symmetry reduction from *Cmcm* to *Pnma* leads to a threefold number of atom sites in the unit cell of (**3**). The difference in molar volumes between (**3**) and (**2**), 6.32 cm^3^/mol, accounts well for the 79% further occupation of the Pt sites, with 79% of the Pt atomic volume of 9.10 cm^3^/mol being 7.19 cm^3^/mol. Why this addition of platinum to Sn_2_Nd affords a more complicated structure is unclear.

**Figure 4:**
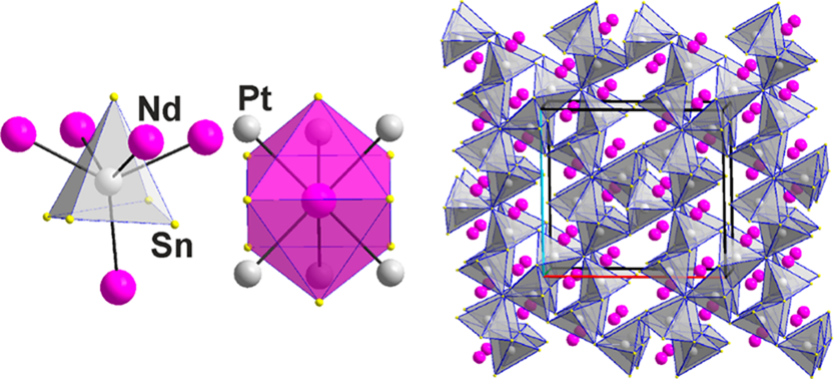
PtSn_2_Nd (**3**). {Pt1Sn_5_Nd_5_} and {Nd1Sn_8_Pt_6_} clusters and the three-dimensional network of {PtSn_5_} polyhedra. Crystallographic axes are color-coded as follows: *a*, red; *b*, green; and *c*, blue.

Examples of typical polyhedra around Pt and Nd, {PtSn_5_Nd_5_} and {NdSn_8–9_Pt_6–5_} in PtSn_2_Nd (3) are shown in Figure 4. Again, interpenetrating {PtSn_5_} and {PtNd_5_} square pyramids with average distances of 2.680(1) and 3.420(1) Å, respectively, are observed, see also Table 2. A view down [010] at the three-dimensional connection of the {PtSn_5_} pyramids is also shown in Figure 4. Although all polyhedra/clusters are nonregular, the surroundings of the three symmetrically independent Nd atoms is more diverse. They include Nd surrounded by either eight (bicapped trigonal prism) or nine (tricapped trigonal prism) Sn atoms, plus six or five Pt atoms in a rather irregular fashion, except for the one surrounding Nd1 in the projection shown in Figure 4, roughly along [001]. Hence, coordination numbers of Pt and Nd are generally 5 + 5 and 14, respectively.

#### Pt_4_Sn_6_Nd_3_ (**4**)

**Figure 5:**
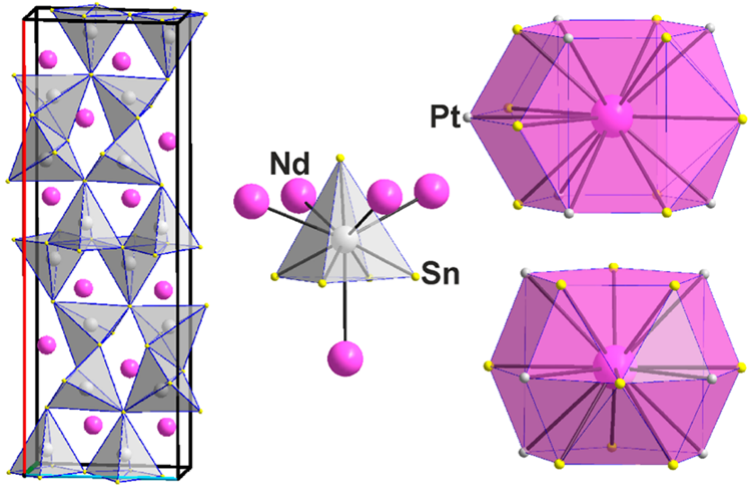
Pt_4_Sn_6_Nd_3_ (**4**). (Left) Three-dimensional network of {PtSn_5_} square pyramids. (Middle) Surrounding of the Pt atoms in the cluster {PtSn_5_Nd_5_} consisting of two interpenetrating Sn_5_ and Nd_5_ square pyramids. (Right) 6–4–6 polyhedron surrounding Nd1 and Nd2, {NdSn_9_Pt_7_}, and 5–5–5 polyhedron surrounding Nd3, {NdSn_8+1_Pt_6_}. Crystallographic axes are color-coded as follows: *a*, red; *b*, green; and *c*, blue.

Pt_4_Sn_6_Nd_3_ (**4**) has, according to Pt_4/3_Sn_2_Nd, a Pt content that is 33.3% higher than in PtSn_2_Nd (**3**). Both (**3**) and (**4**) crystallize with the same space group, *Pnma*, and their lattice constants are related: both have the “usual” short *b* axis, 4.5937(6) (in **3**) and 4.5858(9) Å (**4**), respectively, with cell contents and volumes of Pt_12_Sn_24_Nd_12_ and 1135.52 Å^3^ (**3**) as well as Pt_16_Sn_24_Nd_12_ and 1182.38 Å^3^ (**4**). The difference in molar volumes, 56.99 (**3**) and 59.35 cm^3^/mol (**4**), of 2.36 cm^3^/mol corresponds well with one-third of the volume of a Pt atom, 3.03 cm^3^/mol. In Pt_4_Sn_6_Nd_3_, which crystallizes as Pt_4_Sn_6_R_3_ (R = La, Ce, Pr)^10^ with the Pt_4_Ge_6_Pr_3_ type of structure,^32,33^ all three crystallographically independent Pt atoms form {PtSn_5_} clusters, see Figure 5, with an average Pt–Sn distance of 2.658 (1) Å, nearly the same as in PtSn_2_Nd, 2.680 (1) Å. There are five additional Nd atoms surrounding each Pt such that a {PtSn_5_Nd_5_} heteroatomic cluster of two interpenetrating antiparallel square pyramids occurs (Figure 5), an architecture that we have already seen for all of the stuffed Sn_2_Nd derivatives (**1**), (**2**), and (**3**). For clarity, only the three-dimensional connections of the {PtSn_5_} square pyramids are shown in Figure 5. Pt–Pt distances are all above 4.1 Å and, therefore, well outside the sum of two atomic radii, 2.774 Å, or the sum of the van der Waals radii, 3.50 Å,^34^ regimes. There are three crystallographically independent Nd atoms in the unit cell, two of which, Nd1 and Nd2, exhibit a 6–4–6 coordination sphere, hence CN = 16, and Nd3, which exhibits a 5–5–5 sphere with CN = 15.

#### Pt_2-x_Sn_2+x_Nd (**5**)

**Figure 6:**
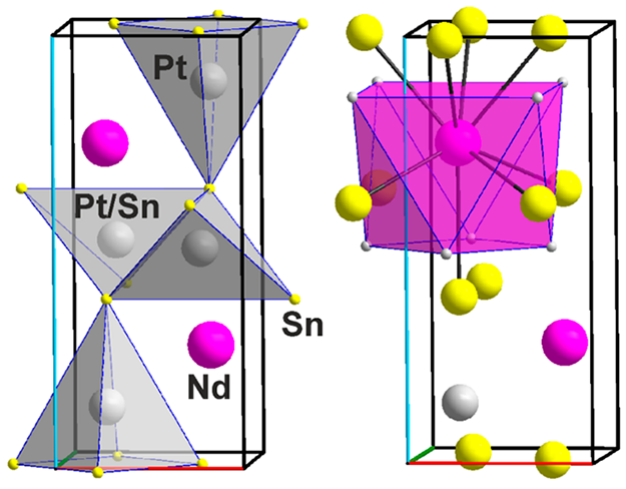
{PtSn_4_} tetrahedra and {PtSn_5_} square pyramids (left) as well as a cluster of a bicapped trigonal prism of Pt atoms and a capped square antiprism of Sn atoms around a Nd atom, {NdPt_8_Sn_9_}, in the crystal structure of Pt_2–*x*_Sn_2+*x*_Nd with *x* = 0.27 (**5**). Crystallographic axes are color-coded as follows: *a*, red; *b*, green; and *c*, blue.

Pt_2–*x*_Sn_2+*x*_Nd (**5**)with *x* = 0.27 crystallizes with the CaBe_2_Ge_2_ type of structure,^35^ Pt_2–*x*_Sn_2+*x*_Nd = Be_2_Ge_2_Ca. The small tetragonal unit cell, *a* = 4.546(4), *c* = 10.409(11) Å contains only two formula units of Pt_2–*x*_Sn_2+*x*_Nd. The molar volume of *V*_*m*_ = 64.78 cm^3^/mol compares well with a normal ∼10% shrinkage relative to the sum of the atomic volumes with the ordered Pt_2_Sn_2_Nd (*V*_*m*_, calc. = 71.36 cm^3^/mol). However, the larger tin atoms substituting 21% of the platinum atoms on the (Pt/Sn)2 site enhance the calculated volume to 73.30%, which is 11.6% larger than the volume of the real unit cell. The mystery becomes even greater when one considers that the larger Sn substitutes the smaller Pt on a tetrahedral site with (Pt/Sn)2–Sn distances of 2.568 (3) Å, which is well beyond the sum of the atomic radii for a weighted (Pt_0.73_/Sn_0.27_)2–Sn distance of 3.038(2) Å. One has to keep in mind, however, that the atomic radii are derived from the structures of the elements for coordination number 12. Furthermore, a comparison of the Pt1–Sn average distance of 2.648(3) Å with CN5 of Pt1 shows that the 2.568(2) Å distance for CN4 is quite reasonable. The Pt–Nd distances are 3.464(3) (Pt1) and 3.398 (3) Å (Pt/Sn2), respectively, also only slightly larger than the sum of the atomic radii, 3.209 Å. Nd is surrounded by a cluster of 17 heteroatoms, {NdPt_8_Sn_9_}, see Figure 6, consisting of a bicapped trigonal prism of 8 Pt atoms and a capped square antiprism of Sn atoms at average distances of 3.431(3) (Nd–Pt) and 3.519(3) (Nd–Sn) Å, which compare fairly well with the sum of the atomic radii for Nd+Pt and Nd+Sn, 3.209 and 3.416 Å, respectively.

#### Pt_3_Sn_2_Nd (**6**)

**Figure 7:**
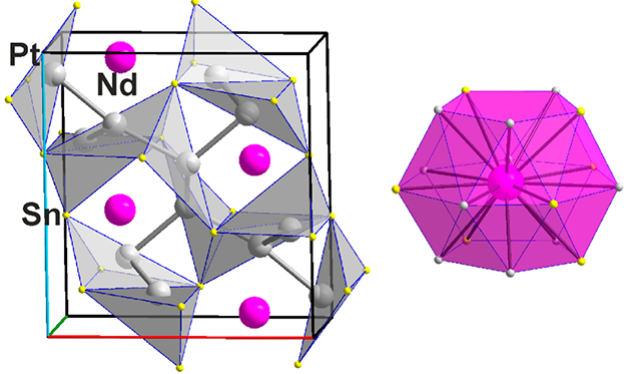
Connection of {PtSn_4_} bisphenoids in the crystal structure of Pt_3_Sn_2_Nd (**6**) together with Pt–Pt “contacts” up to 3.1 Å (left) and the 5–7–5 coordination polyhedron around Nd, {NdPt_10_Sn_7_} (right). Crystallographic axes are color-coded as follows: *a*, red; *b*, green; and *c*, blue.

Pt_3_Sn_2_Nd (**6**) crystallizes with the CePd_3_In_2_/Gd_3_NiSi_2_/Eu_2_CuS_3_ type of structure.^36^ For ternary intermetallics T/E/R, this structure type appears to be quite prolific. Some 20 examples are now known with T = Pd, Pt, E = In, Sn, and R = La–Sm, Sm, Gd, Tb, although not all combinations so far.^12^ Pt_3_Sn_2_Nd is among the compounds already reported and the crystal structure has been refined from single-crystal X-ray data.^12^ With lattice constants of *a* = 9.703 (2), *b* = 4.755 (1), *c* = 1012.8 (3) Å, and *Z* = 4 and space group *Pnma*, the molar volume is *V*_m_ = 70.36 cm^3^/mol, compared to the sum of atomic volumes, 80.46 cm^3^/mol, a rather sharp contraction of 12.6%. Furthermore, the addition of three Pt atoms (3*V*_m_(Pt) = 27.3 cm^3^/mol) to Sn_2_Nd (*V*_m_(Sn_2_Nd) = 48.64 cm^3^/mol, see Table 2) would lead to a molar volume of 75.94 cm^3^/mol, 7.9% larger than the real volume. Thus, the inclusion of Pt into Sn_2_Nd has considerable bonding effects exemplified by the rather short Pt–Sn distances that range from 2.6414 (3) to 2.9221 (8) Å with an average of 2.760 (2) Å, as compared with the sum of the atomic radii of 2.981 Å. Of course, the distances should be shorter as platinum’s coordination number is only four with Sn. The three {PtSn_4_} polyhedra are irregular, far from a tetrahedron, see Figure 7.

With the large number of Pt atoms in the structure, it is not surprising that the Pt–Pt distances are comparatively short, from 2.8444 (6) (Pt2–Pt3) to 3.0847 (4) (Pt3–Pt3) Å, see Figure 7, which are longer than the sum of twice the atomic radius of Pt, 2.774 Å, but considerably shorter than twice the van der Waals radius of Pt, 2 × 1.75 = 3.50 Å.^34^ There is only one crystallographically independent Nd site in the crystal structure of Pt_3_Sn_2_Nd (6); if all Nd–Pt and Nd–Sn distances up to 3.6 Å are considered, a 5–7–5 polyhedron, see Figure 7, with a {NdPt_10_Sn_7_} cluster emerges. Nd–Pt distances range from 3.0153 (6) to 3.5619 (5) Å with an average of 3.268 Å (sum of the atomic radii 3.209 Å) and Nd–Sn distances range from 3.2332 (8) to 3.4797 (6) Å with an average of 3.423 Å (sum of the atomic radii 3.416 Å).

### Intermetallics on or Close to the Pt–Sn_5_Nd_2_ Line

**Figure 8:**
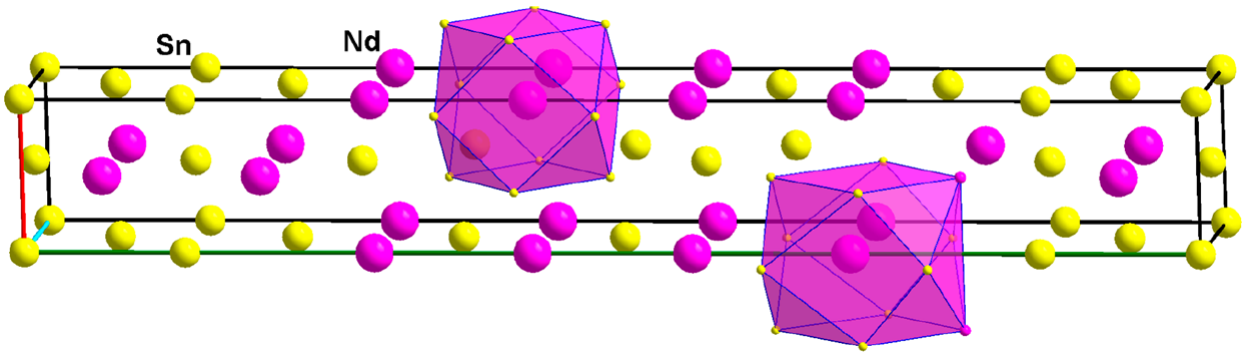
Two mixed-atom surroundings of Nd1 and Nd2 in the crystal structure of Sn_5_Nd_2_, {Nd1@Sn_12_} and {Nd2@Sn_10_Nd_2_}, respectively. Crystallographic axes are color-coded as follows: *a*, red; *b*, green; and *c*, blue.

Sn_5_Nd_2_ crystallizes in the Sn_5_Ce_2_ type^37^ with a sevenfold superstructure of Sn_3_Nd (Cu_3_Au type),^20^ hence with an ordered heteroatomic closest packing of spheres, see Figure 8. The Cu_3_Au type subcells of different compositions stack in the [010] direction. The *b*/*a* = 7.69 and *b*/*c* = 7.61 ratios larger than 7 indicate certain distortion of the core subcell of the Cu_3_Au type and consequently the elongation of the unit cell of Sn_5_Nd_2_ in the *b*-direction.

If there was no under-occupation and disorder of Sn sites in Pt_1.5_Sn_5–*x*_Nd_2_, *x* = 0.16(2) (**7**) and under-occupation of one Nd site in Pt_3_Sn_5_Nd_2-*x*,_ (*x* = 0.161 (**8**)), both intermetallics would lie on the Pt–Sn_5_Nd_2_ section of the ternary Pt/Sn/Nd phase diagram. Pt_3_Sn_5_Nd_2–*x*_ (**8**) had been obtained previously from a reaction of Pt, Sn, and Nd in a molar ratio of 4:6:3 with NaCl added as a nonreactive flux and a maximum reaction temperature of 1000 °C.^10^ At a slightly lower maximum temperature, 900 °C and a ratio of 1:2:1, hence with less Pt, we have now secured Pt_1.5_Sn_5–*x*_Nd_2_ (**7**). (**8**) exhibits an under-occupation at the Nd2 site, 4*c*, SOF = 0.839 (**8**); this accounts for the composition Pt_3_Sn_5_Nd_1.839(8)_ or Pt_3_Sn_5_Nd_2–*x*,_*x* = 0.161. In (**7**), there are two Pt, six Sn and four Nd fully occupied and additional sites under-occupied by Sn, accounting for a total number of atoms in the unit cell of Pt_12_Sn_38.696_Nd_16_, or Pt_3_Sn_9.674_Nd_4_, *Z* = 4, or Pt_1.5_Sn_5–*x*_Nd_2_, *Z* = 8 and *x* = 0.163. With respect to a general formula of Pt_*n*_Sn_5_Nd_2_, it does make sense that the Pt content of (**7**) and (**8**) is *n* = 1.5 and 3, respectively, as less Pt was used as starting material in the reaction that led to (**7**). In (**7**), Nd sites are under-occupied and positionally disordered, and in (**8**) one Nd site is under-occupied but there is no disorder. These observations may be addressed with the usual space-filling and electronic structure arguments.

#### Pt_1.5_Sn_5-x_Nd_2_ (**7**)

**Figure 9:**
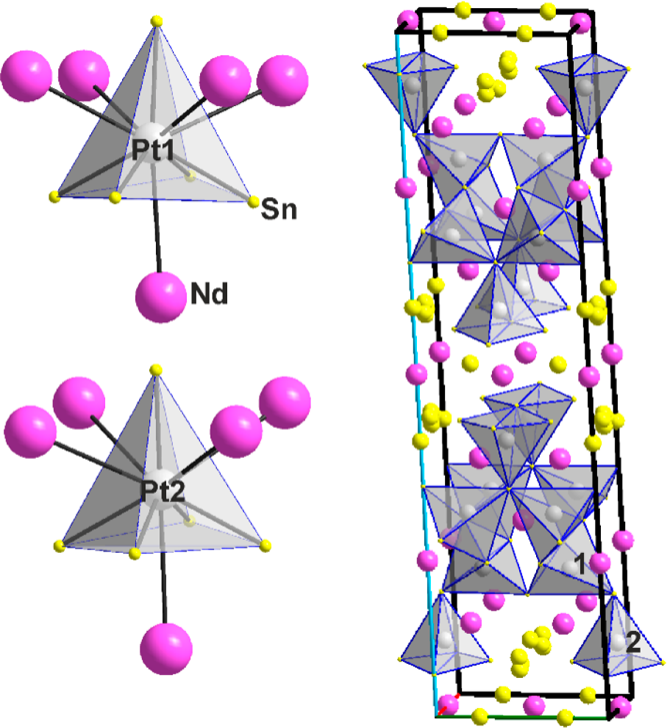
Two crystallographically independent Pt sites in the crystal structure of Pt_3_Sn_10-x_Nd_4_, *x* = 0.326 (**7**), and the unit cell exhibiting the connection of the {PtSn_5_} square pyramids. Crystallographic axes are color-coded as follows: *a*, red; *b*, green; and *c*, blue.

Pt_1.5_Sn_5-x_Nd_2_ (**7**) crystallizes with an unprecedented crystal structure. While the unit cells of Sn_5_Nd_2_ (*a* = 4.5688(6), *b* = 35.119(4), and *c* = 4.6139(6) Å) and Pt_1.5_Sn_5-x_Nd_2_ (*a* = 4.5875(3), *c* = 36.713(3), and *b* = 9.2941(6) Å) are similar, there is no obvious relation between the two structures, see Figures 8 and 9. The increase of the molar volume from Sn_5_Nd_2_ (*V*_m_ = 111.47 cm^3^/mol) to Pt_1.5_Sn_5-x_Nd_2_ (*V*_m_ = 117.85 cm^3^/mol) of only 6.38 cm^3^/mol accounts for roughly only half of the volume 1.5 Pt atoms would afford, 13.65 cm^3^/mol, which is an effect of both filling empty space and chemical bonding. Four Nd, two Pt, and six Sn sites are fully occupied and do not show any metal atom mixing on any of the sites. There is, however, an under-occupation of the Sn7 site and under-occupation and disorder of three further Sn sites, Sn8, Sn9, and Sn10. The latter three are close together, *d*(Sn8–Sn9) = 0.368(3) and *d*(Sn8–Sn10) = 0.657(15) Å, and account for a total of 3.98 Sn atoms, while the Sn7 site accounts for 2.71 Sn atoms. The unit cell content totals to Pt_12_Sn_32+6.696_Nd_16_. Two of the Nd sites are unaffected by this disorder: Nd1 has eight Sn neighbors, from 3.1980(6) to 3.2338(8) Å with an average of 3.209 Å, and six Pt neighbors, from 3.3930(4) to 3.4869(8) Å with an average of 3.424 Å, hence a rather “usual” cluster {Nd1Sn_8_Pt_6_} is observed. Nd4 forms a cluster with the same coordination number, 14, but there are 12 Sn and only 2 Pt atoms the nearest neighbors,

{Nd4Sn_12_Pt_2_}, *d*(Nd4–Sn) = 3.2124(5)–3.4534(7) Å average 3.320 Å, and *d*(Nd4–Pt) = 3.4815(8) and 3.4828(8) Å, average 3.482 Å. The averages compare fairly well with the sum of the atomic radii for Nd + Sn, 3.416 Å, and Nd + Pt, 3.209 Å, with the Nd–Sn averages shrinking and the Nd–Pt averages expanding, a rather usual picture. It is striking that both Pt sites show the formation of {PtSn_5_Nd_5_} clusters, see Figure 9, that we have seen in so many of the Pt_*x*_Sn_2_Nd cases with low Pt content *x*. It is again two antiparallel square pyramids at average distances of 2.683 and 3.369 Å for Pt1–Sn and Pt1–Nd, respectively, as well as 2.625 and of 3.515 Å for Pt2–Sn and Pt2–Nd. For comparison, the sum of the atomic radii equals 2.981 (Pt + Sn) and 3.209 Å (Pt + Nd), hence the Pt–Sn distances are shrinking and the Pt–Nd distances expanding. The connection of the {PtSn_5_} square pyramids as shown in Figure 9 is reminiscent of a layer type structure parallel to the (101) plane and stacking in the [010] direction. Of course, with the Nd atoms added, it is a three-dimensional structure.

#### Pt_3_Sn_5_Nd_1.839(8)_ (**8**)

**Figure 10:**
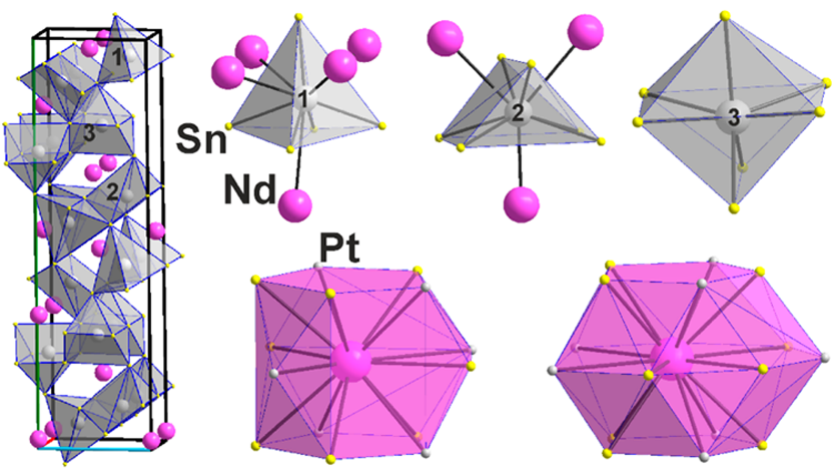
Pt_3_Sn_5_Nd_2–*x*_ (**8**). (Left) Unit cell with the {Pt1Sn_5_}, {Pt2Sn_6_}, and {Pt3Sn_7_} polyhedra connecting. (Upper right) {Pt1Sn_5_Nd_4+1_}, {Pt2Sn_6_Nd_3_}, and {Pt3Sn_7_} clusters. (Lower right) {Nd1Pt_6_Sn_8_} and {Nd2Sn_10_Pt_6_} clusters.

Pt_3_Sn_5_Nd_1.839(8)_ (**8**) crystallizes with the Rh_3_Sn_5_Y_2_ type of structure,^38^ as we have reported in a recently published article.^10^ While in Rh_3_Sn_5_Y_2_ all atomic positions are fully occupied, there is an under-occupation of the Nd2 position in (**8**), 83.9%. This is surprisingly the position with the higher coordination number of Nd, 16, where the average of the 10 Nd2–Sn distances is shorter, 3.330 (4) Å, than the average of the 6 Nd2–Pt distances, 3.510 (4) Å. Thus, a cluster {Nd2Sn_10_Pt_6_} is built, while for Nd1 it is the other way around, {Nd1Pt_6_Sn_8_}, with ⟨*d*(Nd–Pt)⟩ = 3.283 Å (**6**) and ⟨*d*(Nd–Sn)⟩ = 3.327 Å (**8**), see Figure 10. With a higher Pt/Sn_2_Nd_5_ ratio of 3:1, with respect to (**7**), the Pt atoms take over a more dominant structural role. Pt1 exhibits the “usual” {Pt1Sn_5_Nd_5_} cluster, albeit with one rather long Pt–Nd distance of 3.654(4) Å (see the dashed line in Figure 10). Meanwhile, Pt2 has a mixed Sn/Nd surrounding, {Pt2Sn_6_Nd_3_} where the Sn atoms form a distorted trigonal prism, and Pt3 has just seven nearest Sn neighbors, {PtSn_7_} (see Figure 10). These three surprisingly different Pt–Sn–Nd clusters form a three-dimensional network of which only the connections of the Pt–Sn polyhedra are shown in Figure 10. The shortest Pt–Pt distance in (**8**) is 4.284(3) Å, far from twice the van der Waals radius of Pt, 2 × 1.75 = 3.50 Å. Although it is the compound with the highest Pt content in the Pt/Sn_5_Nd_2_ “series”, it has only roughly half the Pt content compared with Pt_3_Sn_2_Nd. Thus, it is no surprise that Pt–Pt “bonding” cannot be attested.

## Conclusions

At present, there are eight ternary intermetallic compounds known in a narrow region of the Pt/Sn/Nd phase space. Pt_0.21(1)_Sn_2_Nd (**2**), PtSn_2_Nd (**3**), Pt_1.33_Sn_2_Nd (**4**), Pt_2–*x*_Sn_2+*x*_Nd (*x* = 0.27(3); **5**), and Pt_3_Sn_2_Nd (**6**) are on or close to the Pt–Sn_2_Nd line, and Pt_1.5_Sn_5–*x*_Nd_2_ (*x* = 0.16(2); **7**) and Pt_3_Sn_5_Nd_2–*x*_ (*x* = 0.161(8); **8**) would be on the Pt–Sn_5_Nd_2_ line if there was no disorder and under-occupation of Sn and Nd sites, respectively. PtNdSn (**1**) is the only known compound in this system situated away from the above ranges. We wonder if similar rows of compounds could be found on the other lines, particularly between Pt and SnNd (in addition to PtSnSn) or other Nd-richer Sn–Nd binary intermetallics.

Crystals of these compounds were obtained from reactions of Pt, Sn, and Nd in 4:6:3 (or 3:6:3) molar ratios with or without the addition of NaCl as a nonreactive flux at maximum temperatures of 1000 °C. Four out of the eight intermetallics were observed for the first time of which (**2**), (**3**), and (**5**) crystallize with known structure types and (**7**) represents a hitherto unknown crystal structure. With an increasing fraction of Pt, we observed for both series a substantial increase in the coordination numbers of Nd (from 12 up to 17) as well as an increasing fraction of Pt in the coordination sphere despite the higher Sn content. Pt atoms strongly prefer square-pyramidal coordination by Sn atoms, resulting in the {PtSn_5_Nd_5_} cluster consisting of two interpenetrating antiparallel square pyramids of Sn and Nd centered by the endohedral Pt atom. Such a cluster perseveres in all structures, even being exclusive for (**2**), (**3**), and (**4**).

## Supporting Information Available

Further information on SEM-EDX and crystallographic information (PDF)

## Dedication

Dedicated to Prof. Dr. Thomas Schleid on the occasion of his 65th birthday.
