# Supplementary material for: The Prolific Ternary System Pt/Sn/Nd: Insertion of Pt into the Structures of Sn/Nd Intermetallics Yields Structural Complexity and Wealth
Source: Inorg Chem. 2023 Jun 2;62(24):9369–78. doi: 10.1021/acs.inorgchem.3c00318 (PMC10283017; doi:10.1021/acs.inorgchem.3c00318)
Supplement: Supplementary file 1 — ic3c00318_si_001.pdf [file ic3c00318_si_001.pdf]

Supporting Information

The Prolific Ternary System Pt/Sn/Nd: Insertion of Pt into the Structures of Sn/Nd Intermetallics Yields Structural Complexity and Wealth

Chris Celania, [a] *Volodymyr Smetana,* [a,b] *Gerd H. Meyer*[c,d*] *and Anja-Verena Mudring* [a,b *]

[a]*Department of Materials and Environmental Chemistry, Stockholm University, Svante Arrhenius väg 16 C, 10691 Stockholm, Sweden.*

[b]*intelligent Advanced Materials, Department of Biological & Chemical engineering and iNANO, 8000 Aarhus C, Denmark.*

[c] *Department of Chemistry, Universität zu Köln, Greinstraße 6, 50939 Köln, Germany.*

[d] *Department of Chemistry, Royal Institute of Technology (KTH), Teknikringen 26, 10042 Stockholm, Sweden.*

Corresponding author emails: gerdm@kth.se; anja-verena.mudring@mmk.su.se; anja-verena.mudring@bce.au.dk

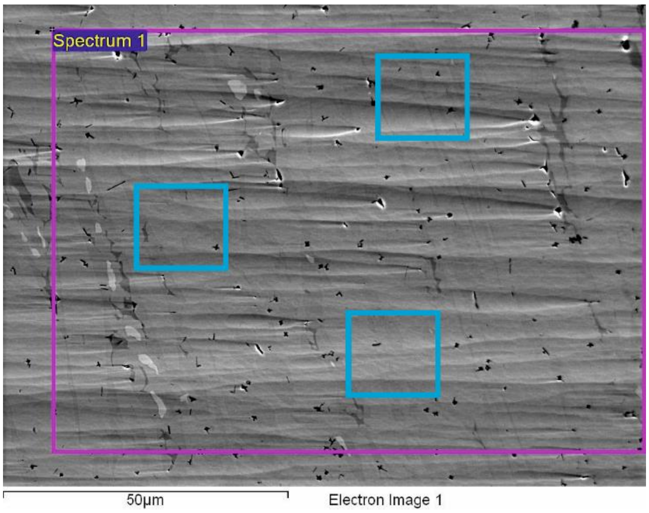

Figure SI. SEM-EDX image of **7**.

Table S1. EDX data for **2**.

Spectrum	Nd	Pt	Sn
1	31.45	5.79	62.76
2	31.00	6.93	62.07
3	30.88	7.20	61.92

Table S2. EDX data for **7**.

Spectrum	Nd	Pt	Sn
1	23.88	17.79	58.33
2	23.05	16.93	60.02
3	23.71	17.59	58.70
